# Reconstruction Error Guided Instance Segmentation for Infrared Inspection of Power Distribution Equipment

**DOI:** 10.3390/s25196007

**Published:** 2025-09-29

**Authors:** Jinbin Luo, Yi Sun, Jian Zhang, Bin Sun

**Affiliations:** 1 CSG Guangdong Power Grid Corporation, Guangzhou 510000, China; 13925959493@139.com (J.L.); xiake-k@163.com (J.Z.); 2 School of Electrical and Information Engineering, Hunan University, Changsha 410082, China; yisun98@hnu.edu.cn; 3 School of Artificial Intelligence and Robotics, Hunan University, Changsha 410082, China

**Keywords:** infrared images, power distribution inspection, instance segmentation, reconstruction error, difference enhancement

## Abstract

The instance segmentation of power distribution equipment in infrared images is a prerequisite for determining its overheating fault, which is crucial for urban power grids. Due to the specific characteristics of power distribution equipment, the objects are generally characterized by small scale and complex structure. Existing methods typically use a backbone network to extract features from infrared images. However, the inherent down-sampling operations lead to information loss of objects. The content regions of small-scale objects are compressed, and the edge regions of complex-structured objects are fragmented. In this paper, (1) the first unmanned aerial vehicle-based infrared dataset PDI for power distribution inspection is constructed with 16,596 images, 126,570 instances, and 7 categories of power equipment. It has the advantages of large data volume, rich geographic scenarios, and diverse object patterns, as well as challenges of distribution imbalance, category imbalance, and scale imbalance of objects. (2) A reconstruction error (RE)-guided instance segmentation framework, coupled with an object reconstruction decoder (ORD) and a difference feature enhancement (DFE) module, is proposed. The former reconstructs the objects, where the reconstruction result indicates the position and degree of information loss of the objects. Therefore, the difference map between the reconstruction result and the input image effectively replays the object features. The latter adaptively compensates for object features by global fusion between the difference features and backbone features, thereby enhancing the spatial representation of objects. Extensive experiments on the constructed and publicly available datasets demonstrate the strong generalization, superiority, and versatility of the proposed framework.

## 1. Introduction

Power distribution equipment is critical infrastructure responsible for delivering low- to medium-voltage electrical power and includes devices such as arresters, breakers, and insulators. Power distribution equipment is susceptible to overheating due to overloaded operations during peak consumption periods (e.g., summer and winter) and prolonged exposure to extreme weather conditions (e.g., icing and typhoons). This can cause equipment failure and may potentially result in widespread power outages. Consequently, regular monitoring of power distribution equipment is essential for ensuring the safe and stable operation of urban power grids [1,2].

The temperature distribution of power distribution equipment can be intuitively visualized in infrared images, as infrared sensors can effectively capture the thermal radiation differences between the power distribution equipment and its surroundings [3,4]. This imaging capability enables accurate temperature measurement and is unaffected by external lighting conditions [5,6].

In the early stages, infrared inspection was typically performed by workers climbing pole towers with handheld infrared cameras to manually examine each piece of power distribution equipment. This method was not only cumbersome and labor-intensive, but also posed significant safety risks, including electrocution and falls from height [7]. In recent years, unmanned aerial vehicles (UAVs) equipped with infrared cameras have been widely adopted due to their high maneuverability and operational flexibility [8,9]. UAVs can easily navigate complex urban environments, such as dense buildings and trees, and observe power distribution equipment from multiple perspectives. Notably, UAVs can cover several kilometers of scenarios in a single operation, significantly improving infrared inspection efficiency.

Instance segmentation, a fundamental task in computer vision, aims at locating the position of each object and recognizing its category within an image [10]. It has become a crucial prerequisite for assessing the operational status of each piece of power distribution equipment [11]. Traditional methods, such as region growing [12], rely on hand-crafted features, such as color and texture. These hand-crafted features are usually designed based on simple image statistics or heuristic rules, which are limited in their representation capabilities. In contrast, modern methods like Mask RCNN [13] have achieved significant advancements by automatically learning complex and nonlinear spatial and semantic relationships between foreground objects and background from large-scale images. As a result, modern methods have been successfully transferred from visible images of natural scenarios to infrared images of power scenarios [14].

The challenge of accurately locating and recognizing power distribution equipment in images stems from the following reasons. Firstly, power distribution equipment, such as a bushing, is physically small, and when imaged from an operational distance by UAVs, it occupies a minuscule number of pixels, making it a small-scale object. Secondly, power distribution equipment, like an insulator or a disconnector, possesses intricate geometries, rendering it a complex-structured object. Therefore, due to these specific characteristics, power distribution equipment is generally characterized by small scale and complex structure. Meanwhile, due to the hardware limitations of infrared sensors, the resolution of infrared images is low. In this situation, the above challenge becomes even more severe.

**Figure 1:**
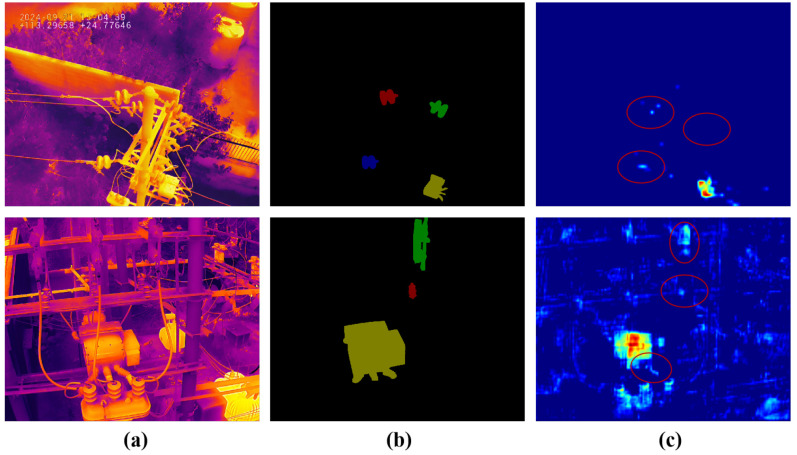
Schematic diagram illustrating the information loss caused by down-sampling operations in the backbone network. (**a**) Input infrared image; (**b**) ground truth; (**c**) feature maps from the 4th and 1st levels of the backbone network. The content regions of small-scale objects are compressed, with their features nearly erased, while the edge regions of objects with complex structures are discretized, resulting in fragmented features, as highlighted by the red circles.

Modern methods typically employ backbone networks to extract hierarchical features from infrared images. However, the inherent down-sampling operations in backbone networks lead to information loss of objects, as illustrated in Figure 1. Specifically, the content regions of small-scale objects collapse, i.e., decrease or even disappear. Their features can be reduced from several pixels to a single pixel or even less, which significantly compresses their content information, making them indistinguishable from noise or background. The edge regions of objects with complex structures are discretized, i.e., jagged and fragmented. The discretization process causes a sharp and continuous edge to become a jagged, broken series of disconnected points, fragmenting the structural integrity of the object’s boundary. Therefore, missed recognition and coarse localization of objects frequently occur in modern methods.

To control the information loss of objects, super resolution (SR) [15,16] and improved down-sampling (IDS) operations [17,18] are considered effective approaches. Specifically, SR reconstructs high-resolution (HR) counterparts from low-resolution images. Although super-resolution increases the information capacity of objects by enhancing pixel density, it is susceptible to generating fake textures and undesirable artifacts. Furthermore, HR images increase the computational burden of models. In contrast, IDS performs a weighted summation of all pixels within the sampling region to aggregate their information. However, this process can be laborious.

To resolve the above problems, a reconstruction error (RE)-guided instance segmentation framework is proposed, whose core idea is to replay and compensate for object features to enhance their spatial feature representations. The framework consists of two main components.

Specifically, (1) an object reconstruction decoder (ORD) is proposed to reconstruct the object regions from the backbone features. The reconstruction result indicates the position and degree of information loss of objects. Correspondingly, the difference map (reconstruction error map) between the reconstruction result and the input image effectively replays the object features. (2) A difference feature enhancement (DFE) module is proposed, which adaptively compensates for object features through global interaction and fusion between the difference features and the backbone features, thereby enhancing the spatial features of objects. The ORD and the DFE module are constrained by the pixel-wise error between the reconstruction result and the input image, which is sensitive to fine-grained pixel changes of objects and possesses self-supervision capability. (3) A real-world infrared dataset, PDI, consisting of 16,596 images, 126,570 (120K+) instances, and 7 critical categories of power equipment is constructed. The dataset covers scenarios from hundreds of geographic regions and object patterns from multiple observation viewpoints. Compared to existing infrared datasets, PDI is the first UAV-based infrared dataset for distribution inspection and has the largest data volume, rich scenarios, and diverse object patterns. Meanwhile, this dataset poses challenges of scale imbalance, category imbalance, and distribution imbalance of objects.

The proposed framework effectively captures small-scale objects and preserves the edge integrity of objects with complex structures, providing insight into the scale imbalance problem. Extensive experiments on both the constructed and publicly available datasets demonstrate the superiority, generalization, and versatility (model-agnostic) of the proposed framework, thereby greatly advancing power distribution inspection. Our code is publicly available on https://github.com/yisun98 (accessed on 23 September 2025).

The contributions of this paper can be summarized as follows:

- The first real-world UAV-based infrared dataset for distribution inspection is proposed. This dataset offers the largest data volume, diverse geographic scenarios, and varied instance patterns, while also presenting challenges such as scale imbalance, distribution imbalance, and category imbalance. It establishes a comprehensive performance benchmark and validates the superiority of the proposed framework.
- An object reconstruction mechanism is proposed to effectively mitigate the information loss of objects by reconstructing the object regions. This approach reveals the correlation between reconstruction and degradation, providing effective object features via reconstruction error.
- A difference feature enhancement (DFE) module is proposed to enhance the spatial feature representations of objects, effectively preserving both the content integrity and the edge continuity of objects.

The remainder of this paper is organized as follows. Section 2 briefly reviews instance segmentation methods. Section 3 describes the proposed framework in detail. Section 4 introduces the constructed dataset. Section 5 provides experiments and analysis to explore the limitations of existing methods and the working mechanism of the core components. Section 6 discusses the complexity and application scenarios of the proposed framework. Section 7 gives conclusions and points out future work.

## 2. Related Work

### 2.1. General Instance Segmentation

Traditional Methods: Region growing is a representative traditional method [12]. It begins by randomly selecting several seed pixels as starting points, and then progressively merges neighboring pixels based on a defined similarity measure to form connected regions, with each region corresponding to a distinct object. The similarity between pixels can be determined by features such as color, texture, and gradient. Notably, region growing is sensitive to gradients, enabling it to effectively capture object boundaries. However, this method is prone to generating isolated or fragmented regions in the presence of noise. More broadly, traditional methods like region growing heavily rely on hand-crafted features, which are typically designed using basic image statistics or simple heuristic rules [10]. As a result, their feature representation capabilities are inherently limited, often leading to performance bottlenecks in complex or dynamic scenarios. Furthermore, the design of hand-crafted features requires domain-specific knowledge, introducing human intervention bias and restricting generalization ability.

Modern Methods: In recent years, deep convolutional neural networks (DCNNs) have emerged as the mainstream solution. This can be attributed to their ability to automatically learn complex and nonlinear spatial and semantic relationships between foreground objects and backgrounds from large-scale image datasets, significantly enhancing performance. Modern methods can be categorized into two-stage and one-stage approaches. Two-stage methods, primarily based on the RCNN family, employ a region proposal network (RPN) to generate candidate object regions. The RPN operates on each pixel of the feature map using predefined anchors of different aspect ratios, thereby reducing the likelihood of missed detections. Representative two-stage methods include Mask RCNN [13] and Cascade RCNN [19]. In contrast, one-stage methods, based on the SOLO and YOLO families, can directly predict object instances. For example, the SOLO family divides the feature map into a grid of cells, with each cell responsible for predicting an object if its center falls within that grid. Representative one-stage methods include SOLOv2 [20] and YOLOACT [21].

### 2.2. Instance Segmentation of Power Infrared Scenarios

In recent years, both two-stage and one-stage modern methods have achieved remarkable performance on visible images of natural scenarios [8]. Transfer learning is a technique that allows models to generalize knowledge learned in a source domain to a target domain, achieving competitive performance. With the support of transfer learning, modern methods have been increasingly applied to infrared images of power scenarios, leveraging knowledge learned from visible domains to improve performance in infrared domains.

For example, Zhao et al. [22] presented category-specific image patches as distinct spatial priors to better distinguish visually similar objects. Zhou et al. [14] proposed a shuffle-polarized self-attention module to capture long-range spatial dependencies, thereby enhancing objects with complex structures. Li et al. [8] constructed a multi-task learning framework that jointly performs super-resolution and instance segmentation, facilitating the transfer of high-resolution spatial texture and details to improve object discriminability. Li et al. [23] presented a spatial transformation network to extract the multi-scale spatial context of objects. These studies collectively demonstrate that state-of-the-art modern methods are effective for power infrared scenarios. However, information loss of objects can still lead to relatively coarse results.

## 3. Methodology

### 3.1. Overall Architecture

**Figure 2:**
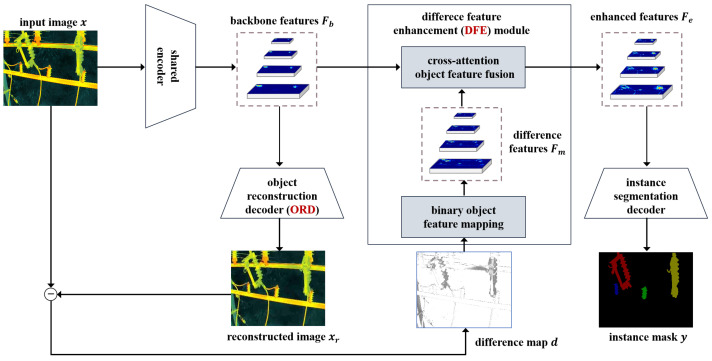
Overall architecture of the proposed framework.

The overall architecture of the proposed framework is illustrated in Figure 2, which consists of four parts:

(1) Shared Encoder $\Psi_{\mathrm{enc}}$: A backbone network, such as ResNet, pretrained on large-scale datasets (e.g., ImageNet), coupled with a neck network, such as FPN [24], is employed to extract multi-scale backbone features $F_{b}=\left\{f_{l}\in R^{H/2^{l}\times W/2^{l}\times C}|l=1,2,\ldots ,L\right\}$ from the input infrared image $x\in R^{H\times W\times 3}$:

(1) $$F_{b}=\Psi_{\mathrm{enc}}\left(x\right)$$

where *H* and *W* denote the height and width of the image, *C* is the number of feature channels, and *l* indicates the feature level. Importantly, the encoder is shared across both the object reconstruction decoder and the instance segmentation decoder, ensuring feature consistency.

In general, the backbone network involves inherent down-sampling operations, typically implemented by max-pooling layers or convolutional layers with a stride greater than one [17]. These down-sampling operations are essential for constructing hierarchical feature representations and expanding the receptive field. However, they inevitably result in information loss of objects, where the content regions of small-scale objects are compressed (e.g., diminished or even lost), and the edge regions of objects with complex structures are discretized (e.g., fragmented and jagged), making their features less distinguishable.

(2) Object Reconstruction Decoder $\Psi_{\mathrm{rdec}}$: The image $x_{r}\in R^{H\times W\times 3}$ is reconstructed from the features $F_{b}$ such that it approximates the original input image x as closely as possible:

(2) $$x_{r}=\Psi_{\mathrm{rdec}}\left(F_{b}\right)$$

This process follows a self-supervised learning paradigm, where the input image x itself serves as the supervision signal. The reconstruction is object-centric: object regions are preserved with their original pixel values, while non-object regions are suppressed to zero. Correspondingly, the reconstruction result explicitly reflects the position and degree of information loss of objects. Moreover, the reconstruction is constrained by the pixel-wise error between it and the input image, revealing fine-grained pixel-level details of objects.

(3) Difference Feature Enhancement Module $\Psi_{\mathrm{st}}$: The difference map (reconstruction error map) $d\in R^{H\times W\times 3}$ between the reconstructed image and the input image is used as input. This map describes spatial discrepancies in object regions, effectively replaying the object features. The difference feature enhancement module extracts difference features $F_{d}=\left\{f_{m}\in R^{H/2^{l}\times W/2^{l}\times C}|l=1,2,\ldots ,L\right\}$, and adaptively compensates for object features through a global interaction mechanism between the backbone features $F_{b}$ and the difference features:

(3) $$F_{e}=\Psi_{\mathrm{st}}\left(F_{b},F_{d}\right)$$

(4) Instance Segmentation Decoder $\Psi_{\mathrm{sdec}}$: The final instance-level prediction is generated through three parallel branches: a classification branch for recognizing object categories, a regression branch for locating objects via bounding boxes, and a segmentation branch for delineating object boundaries [13]. These branches share the enhanced spatial features $F_{e}$ as input. The prediction y is supervised using the corresponding instance masks associated with the input infrared image x:

(4) $$y=\Psi_{\mathrm{sdec}}\left(F_{e}\right)$$

where y includes the predicted category set c, bounding box set b, and binary mask set m. c contains the highest probability category for each object, b consists of the coordinates of the upper-left and lower-right corners of each bounding box $\left[p_{x},p_{y},q_{x},q_{y}\right]$, and m represents the binary mask for each object.

The proposed framework is explicitly designed to mitigate information loss of power distribution equipment, reflecting its specific characteristics. For content compression of small-scale objects, the ORD counteracts loss by enforcing the model to learn features capable of reconstruction. The DFE module then takes the difference map between the reconstructed result and the input image to re-inject these features, effectively compensating for lost content and enhancing focus on small regions. For edge fragmentation of objects with complex structures, the object reconstruction loss acts as a per-pixel spatial constraint, which is highly sensitive to errors along edges and fine details. Consequently, the ORD learns object features that accurately reconstruct complex edges, and the resulting difference map serves as a self-supervised attention map highlighting edge regions. The DFE module’s fusion mechanism uses this attention map to adaptively sharpen and enhance feature representations along object boundaries, restoring structural continuity.

### 3.2. Object Reconstruction Decoder

**Figure 3:**
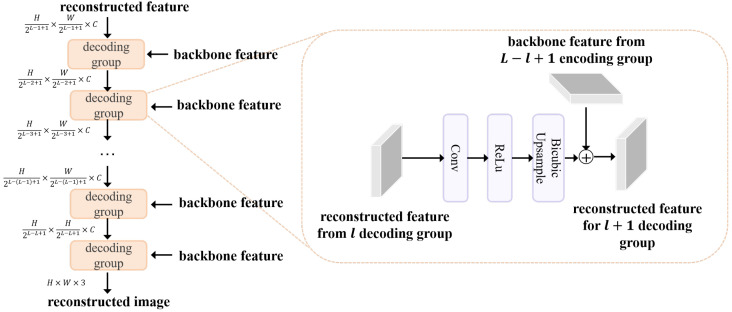
Architecture of the ORD, which consists of multiple decoding groups. The detailed structure and feature dimension of each decoding group are presented.

The information loss of objects makes it difficult to preserve sufficient features for reconstructing their original morphology. The object reconstruction decoder (ORD) is proposed to reconstruct object regions from the backbone features, where the reconstruction result reflects the position and degree of information loss of objects. We present the architecture of ORD in Figure 3.

In general, the ORD adopts a symmetric architecture with respect to the shared encoder. Each decoding group comprises a 3×3 convolutional layer, a ReLU activation, and a bicubic interpolation operation. These layers serve to reduce feature channels, nonlinearly activate features, and upsample features. The decoding process is defined as:

(5) $$\begin{array}{c} f_{m+1}=\mathrm{Bicubic}\left(\mathrm{ReLU}\left({\mathrm{Conv}}_{3\times 3}\left(f_{m}\right)\right),\mathrm{scale}=2\right)+f_{l} \end{array}$$

where $f_{m+1}$ is the output feature, l=1,2,…,L is the feature level, and m=L−l+1. The input and output channels of the convolutional layer are both *C*, except for the last layer, which has an output channel of 3. After *L* decoding processes, the reconstructed image $x_{r}$ is generated, maintaining the same resolution as the input image x.

The bicubic interpolation operation [16] is a standard method for up-sampling images or feature maps, providing smoother results than nearest-neighbor or bilinear interpolation. The formula for bicubic interpolation is given by:

(6) $$f\left(x,y\right)=\sum_{i=-1}^{2}\sum_{j=-1}^{2}h\left(x-i\right)h\left(y-j\right)f\left(x_{0}+i,y_{0}+j\right)$$

(7) $$h\left(t\right)=\left\{\begin{array}{cc} {\left(a+2\right)|t|}^{3}-\left(a+3\right){\left|t\right|}^{2}+1, & 0\leq |t|<1,\\ {a|t|}^{3}-{5a|t|}^{2}+8a\left|t\right|-4a, & 1\leq |t|<2,\\ 0, & |t|\geq 2, \end{array}\right.$$

where f(x,y) is the interpolated feature value at the continuous coordinate (x,y), $f(x_{0}+i,y_{0}+j)$ is the feature value at integer grid point $\left(x_{0}+i,y_{0}+j\right)$, $\left(x_{0},y_{0}\right)$ is the integer part of coordinate (x,y), i,j∈{−1,0,1,2} are offsets indicating the 4×4 neighborhood around $\left(x_{0},y_{0}\right)$, h(t) is the cubic convolution kernel, and *a* is a kernel parameter.

Compared to existing SR-based and IDS-based methods, the object reconstruction mechanism has three distinctive advantages: (1) Object-centric reconstruction: Unlike SR, which reconstructs the entire image including uninformative background, this mechanism guides the model to focus reconstruction on informative object regions. This is a more efficient approach. When calculating the reconstruction loss, the object regions are preserved with their original pixel values, while non-object regions are suppressed to zero. This masking strategy also enhances the model’s sensitivity to object regions. (2) Pixel change sensitivity: Unlike IDS, the reconstruction process is constrained by per-pixel errors between the reconstruction result and the input image. The reconstruction loss provides a strong, fine-grained gradient signal for every pixel, making the output highly responsive to even minor pixel value changes. (3) Self-supervised learning: The supervision signal (the original image) is freely available and requires no additional external data or manual annotation (e.g., high-resolution images needed by SR). Furthermore, SR is susceptible to generating fake textures and artifacts, and HR images increase the computational burden. This mechanism is therefore more practical.

### 3.3. Difference Feature Enhancement Module

**Figure 4:**
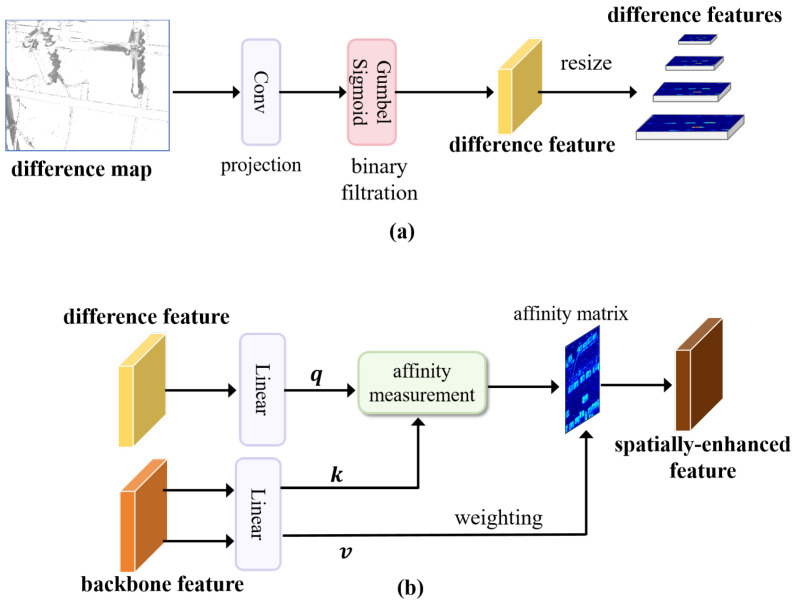
Architecture of the DFE module. (**a**) Projection and filtration, used for binary object feature mapping; (**b**) embedding, used for cross-attention object feature fusion.

The difference map (reconstruction error map) between the reconstruction result and the input image explicitly replays object prior clues. Therefore, a difference feature enhancement (DFE) module (Figure 4) is proposed. It projects the difference map from the image space into the feature space, applies binary filtering with the Gumbel-sigmoid function to suppress background noise, and finally adaptively compensates object-related clues via global interaction and fusion between the difference features and the backbone features. In this way, the spatial feature representations of objects are effectively enhanced.

The DFE module consists of the following three steps:

(1) Projection and filtration for binary object feature mapping. The difference map $d\in R^{H\times W\times 3}$ is generated by subtracting the reconstructed image $x_{r}$ from the input image x and taking the absolute value:

(8) $$d=\mathrm{Abs}(x_{r}-x)$$

where Abs denotes the element-wise absolute value. A 3×3 convolutional layer is then applied to project the difference map d from the low-dimensional image space into the high-dimensional feature space:

(9) $$f_{m}={\mathrm{Conv}}_{3\times 3}\left(d\right)$$

where $f_{m}$ is the difference feature, and the numbers of input and output channels are 3 and *C*, respectively.

Due to pixel differences between $x_{r}$ and x, the difference map tends to be activated at nearly every pixel, introducing significant background noise. To address this, a Gumbel-sigmoid function [25] is applied to generate a binary mask ${\hat{f}}_{m}$, highlighting object regions:

(10) $${\hat{f}}_{m}=\frac{1}{1+\exp\left(-\frac{1}{\tau }\left(f_{d}+G_{1}-G_{2}\right)\right)}$$

where τ is a temperature parameter controlling the sharpness of the approximation, and $G_{1},G_{2}∼\mathrm{Gumbel}\left(0,1\right)$ are independent samples from the Gumbel distribution. This function retains differentiability, ensuring foreground-background separation and enhancing object contrast.

(2) Embedding for cross-attention feature fusion. To align the difference features with the backbone features at multiple scales, bicubic interpolation is applied to resample ${\hat{f}}_{d}$ with scale factors $\left\{2^{1},2^{2},\ldots ,2^{l}\right\}$:

(11) $$F_{d}=\left\{\mathrm{Bicubic}\left({\hat{f}}_{d},\mathrm{scale}=2^{l}\right),\;l=1,2,\ldots ,L\right\}$$

where the scale factor aligns with the ratio of adjacent levels in the backbone feature hierarchy.

After alignment, taking features of any scale as an example, a set of linear transformations is used to produce the query q from the difference feature ${\hat{f}}_{d}$, and the key k and value v from the backbone feature $f_{b}$:

(12) $$q=\mathrm{Linear}\left({\hat{f}}_{d}\right),\;k=\mathrm{Linear}\left(f_{b}\right),\;v=\mathrm{Linear}\left(f_{b}\right)$$

where Linear denotes a fully connected layer.

The cross-attention fusion is then modeled as global feature affinity measurement and weighting:

(13) $$f_{e}=\left(\frac{\exp\left(q\cdot k^{T}/\sqrt{s}\right)}{\sum_{j}\exp\left(q\cdot k_{j}^{T}/\sqrt{s}\right)}\right)\cdot v$$

where *T* denotes matrix transpose, and $\sqrt{s}$ is a scaling factor to normalize dot products, set to 0.5. The resulting enhanced feature $f_{e}$ captures spatially compensated feature representations of objects.

The DFE module has the following advantages: (1) Global spatial awareness: Object-related clues ${\hat{f}}_{m}$ are re-injected into the backbone features through global (pixel-to-pixel) interaction and fusion, restoring degraded object features. (2) Plug-and-play capability: The enhanced features $f_{e}$ can be seamlessly integrated into existing one-stage and two-stage instance segmentation decoders. They improve the content integrity of small-scale objects and preserve edge continuity for objects with complex structures.

### 3.4. Objective Function

The objective function of the proposed framework consists of the object reconstruction loss and the instance segmentation loss.

(1) Object Reconstruction Loss $L_{\mathrm{OR}}$: The object reconstruction loss is defined as the pixel-wise mean absolute error (MAE) [26] between the reconstructed image $x_{r}$ and the input image x:

(14) $$L_{\mathrm{OR}}={∥x_{r}-x∥}_{1}$$

where ${∥\cdot ∥}_{1}$ denotes the $l_{1}$-norm. Importantly, $L_{\mathrm{OR}}$ is computed only within object regions and can be regarded as a variant of *deep supervision*. Compared with the mean squared error (MSE), the MAE loss is less sensitive to large outliers, which is advantageous for image reconstruction. It yields sharper results and is more tolerant of occasional bright pixels (e.g., light reflections) that would otherwise dominate the gradient of the MSE loss.

(2) Instance Segmentation Loss $L_{\mathrm{IS}}$: *Without loss of generality*, the instance segmentation loss consists of a classification loss $L_{c}$, a regression loss $L_{r}$, and a segmentation loss $L_{s}$ [13]. Specifically, $L_{c}$ determines the category of each object, $L_{r}$ determines the bounding box of each object, and $L_{s}$ determines the mask of each object. The overall formulation is:

(15) $$\begin{array}{cc} L_{\mathrm{IS}} & =L_{c}\left(c,c^{*}\right)+L_{r}\left(b,b^{*}\right)+L_{s}\left(s,s^{*}\right) \end{array}$$

(16) $$\begin{array}{cc} & =-\sum_{i=1}^{N}c_{i}^{*}\log\left(c_{i}\right)+\sum_{i=1}^{4}{\mathrm{smooth}}_{L1}\left(b_{i}-b_{i}^{*}\right) \end{array}$$

(17) $$\begin{array}{cc} & \;+\sum_{i=1}^{H}\sum_{j=1}^{W}\left[s_{i,j}^{*}\log\left(s_{i,j}\right)+\left(1-s_{i,j}^{*}\right)\log\left(1-s_{i,j}\right)\right] \end{array}$$

where c and $c^{*}$ are the predicted and ground-truth categories, b and $b^{*}$ are the predicted and ground-truth bounding boxes, and s and $s^{*}$ are the predicted and ground-truth masks. In practice, $L_{c}$ and $L_{s}$ are implemented with the cross-entropy loss, while $L_{r}$ is computed with the smooth L1 loss [27]. The cross-entropy loss is the standard choice for multi-class classification and pixel-level segmentation, as it penalizes the divergence between predicted and actual distributions, thereby encouraging high confidence for the correct category and suppressing incorrect predictions. The smooth L1 loss, combining the advantages of MAE and MSE, behaves like MSE for small residuals (ensuring stable gradients) and like MAE for large residuals (enhancing robustness to outliers), making it highly effective for bounding box regression.

(3) Total Loss: The overall training objective is defined as:

(18) $$L_{\mathrm{total}}=L_{\mathrm{IS}}+\lambda L_{\mathrm{OR}}$$

where λ is a weighting factor to balance the two losses. In this work, λ is set to 1.0, assigning equal importance to both reconstruction and segmentation objectives.

### 3.5. Training and Testing Pipelines

We define the input, output, and detailed intermediate steps of the proposed framework in Algorithms 1 and  2.
**Algorithm 1** Training pipeline of RE-guided instance segmentation framework**Input:**- Input infrared image x- Ground truth instance segmentation mask $y^{*}$ (includes category $c^{*}$, bounding box $b^{*}$, mask $s^{*}$, for each power distribution equipment)**Output:**- Predicted instance segmentation result y includes category c, bounding box b, mask s, for each power distribution equipment)- Reconstructed infrared image $x_{r}$**Steps:**1: Extract multi-scale backbone features: $F_{b}=\Psi_{\mathrm{enc}}\left(x\right)$ *// Equation (1)*2: Reconstruct the image from multi-scale backbone features: $x_{r}=\Psi_{\mathrm{rdec}}\left(F_{b}\right)$ *// Equation (2)*3: Compute the reconstruction error (difference map): $d=\mathrm{Abs}(x_{r}-x)$ *// Equation (6)*4: Project and filter difference map to obtain object features ${\hat{f}}_{d}$ *// Equations (7) and (8)*5: Align object features with backbone features to get multi-scale difference features $F_{d}$ *// Equation (9)*6: Fuse multi-scale backbone and difference features: $F_{e}=\Psi_{\mathrm{st}}\left(F_{b},F_{d}\right)$ *// Equations (10) and (11)*7: Predict instance segmentation mask from multi-scale fused features: $y=\Psi_{\mathrm{sdec}}\left(F_{e}\right)$ *// Equation (4)*8: Compute object reconstruction loss: $L_{\mathrm{OR}}=\left|\right|x_{r}-x{\left|\right|}_{1}$ *// Equation (12)*9: Compute instance segmentation loss: $L_{\mathrm{IS}}=L_{c}\left(c,c^{*}\right)+L_{r}\left(b,b^{*}\right)+L_{s}\left(s,s^{*}\right)$ *// Equations (13)–(15)*10: Update network parameters by minimizing the total loss: $L_{\mathrm{total}}=L_{\mathrm{IS}}+\lambda L_{\mathrm{OR}}$ *// Equation (16)***Return:** y, $x_{r}$

**Algorithm 2** Testing pipeline of RE-guided instance segmentation framework

**Input:**

- Input infrared image $x_{\mathrm{test}}$

**Output:**

- Predicted instance segmentation result $y_{\mathrm{pred}}$ (includes category c, bounding box b, mask s, for each power distribution equipment)

**Steps:**

1: Extract multi-scale backbone features: $F_{b}=\Psi_{\mathrm{enc}}\left(x_{\mathrm{test}}\right)$ *// Equation (1)*
2: Reconstruct infrared image from multi-scale backbone features: $x_{r}=\Psi_{\mathrm{rdec}}\left(F_{b}\right)$ *// Equation (2)*
3: Compute the reconstruction error (difference map): $d=\mathrm{Abs}(x_{r}-x_{\mathrm{test}})$ *// Equation (6)*
4: Project and filter difference map to obtain object features ${\hat{f}}_{d}$ *// Equations (7) and (8)*
5: Align object features with backbone features to get multi-scale difference features: $F_{d}$ *// Equation (9)*
6: Fuse multi-scale backbone and difference features: $F_{e}=\Psi_{\mathrm{st}}\left(F_{b},F_{d}\right)$ *// Equations (10) and (11)*
7: Predict instance segmentation mask from multi-scale fused features: $y_{\mathrm{pred}}=\Psi_{\mathrm{sdec}}\left(F_{e}\right)$ *// Equation (4)*
**Return:** $y_{\mathrm{pred}}$


## 4. Dataset Construction

### 4.1. Image Acquisition

The UAV platforms used for image acquisition are primarily manufactured by DJI, including models such as the Mavic and Air series. These UAVs are equipped with uncooled vanadium oxide (VOx) microbolometer radiometric infrared cameras, which operate based on passive thermal imaging. The infrared cameras provide a thermal sensitivity of less than 50 mK@f/1.0 and cover a spectral wavelength range of 8∼14 μm. Infrared images are recorded in R-JPEG* format (16-bit) with a spatial resolution of 640 × 512 pixels. The cameras are further fitted with lenses offering a diagonal field of view (DFOV) of 40.6° and a focal length of 13.5 mm.

The temperature effect in infrared imaging may influence subsequent overheating analysis. To address this, the infrared cameras are equipped with hardware-level compensation and an internal calibration mechanism. Specifically, ambient temperature variations affecting the sensor are mitigated through an internal shutter-based non-uniformity correction (NUC). During flight, the cameras periodically initiate a calibration cycle in which the shutter briefly blocks the sensor to measure and correct for thermal drift. In addition, each infrared image contains embedded pixel-wise radiometric temperature metadata, which can be extracted using standard third-party thermal analysis tools. This ensures that the recorded imagery remains reliable for subsequent temperature-related overheating analysis.

With the support of the China Southern Power Grid (CSG), infrared data acquisition is carried out by workers from the local power supply bureau. The power distribution equipment is observed under operating conditions.

### 4.2. Equipment Categories and Functional Roles

Within distribution systems, each type of equipment fulfills a critical role in ensuring system safety and reliability. Specifically, bushings enable the safe passage of conductors through transformer tanks while providing electrical insulation. Insulators deliver essential mechanical support and electrical isolation for overhead lines and substation equipment. Disconnectors provide visible isolation points in circuits, facilitating safe maintenance operations. Arresters protect equipment from voltage surges induced by lightning strikes or switching events. Breakers interrupt fault currents and isolate faulty sections of the network. The switch offers circuit protection by interrupting overcurrent conditions through fuse operation. Finally, terminal ensures reliable and insulated connections between power cables and associated equipment.

All of the aforementioned power distribution equipment belongs to the distribution network tier. The operating voltage primarily falls within the medium- or low-voltage range (e.g., 10 kV, 35 kV).

### 4.3. Image Annotation and Extension

(1) Category: Each object is assigned a unique category ID based on a fixed mapping scheme. The dataset covers seven critical categories of power distribution equipment: bushing, insulator, disconnector, arrester, breaker, switch, and terminal.

(2) Instance Mask: Each object is annotated with dense boundary points to form a polygonal mask, and the enclosed region is taken as its corresponding instance-level mask. The number of points ranges from tens to hundreds, depending on the object’s structural complexity and category. Truncated objects are annotated only if the visible portion exceeds 60% of the original object size. All annotations strictly follow the Pascal COCO standard, with each infrared image paired with a corresponding JSON annotation file. In total, 16,596 infrared images are annotated, containing 126,570 (120K+) instances. Following a predefined 8:2 ratio, the dataset is split into training and testing subsets, containing 13,276 and 3320 images, respectively, with 107,486 and 23,484 annotated instances.

(3) Annotation Extension for Other Downstream Tasks: For semantic segmentation, annotations are generated without distinguishing individual objects within the same category. For object detection, annotations are defined as the minimum enclosing bounding rectangles of each object, represented by the coordinates of their four vertices.

(4) Annotation Quality Control: To ensure annotation consistency and dataset reliability, the following strategies were adopted:

- Annotation team: Seven professionally trained annotators from a data service company conducted the labeling, having received prior instruction on the specific categories of power distribution equipment.
- Annotation guidelines: A detailed guideline document was prepared, including category definitions, correct/incorrect annotation examples, and rules for handling special cases (e.g., occlusion and truncation).
- Quality supervision: Two senior engineers from China Southern Power Grid supervised the entire process. They continuously reviewed randomly selected subsets from each annotator. Ambiguities were discussed and resolved collectively, with iterative updates to the guidelines.
- Inter-annotator agreement: A randomly selected subset was cross-annotated by different annotators, and the average precision between the two sets of annotations was calculated. A high score quantitatively confirmed annotation consistency.

### 4.4. Comparison with Existing Infrared Datasets

Table 1 provides a comparative analysis between the proposed infrared dataset (PDI) and existing infrared datasets.

**Figure 5:**
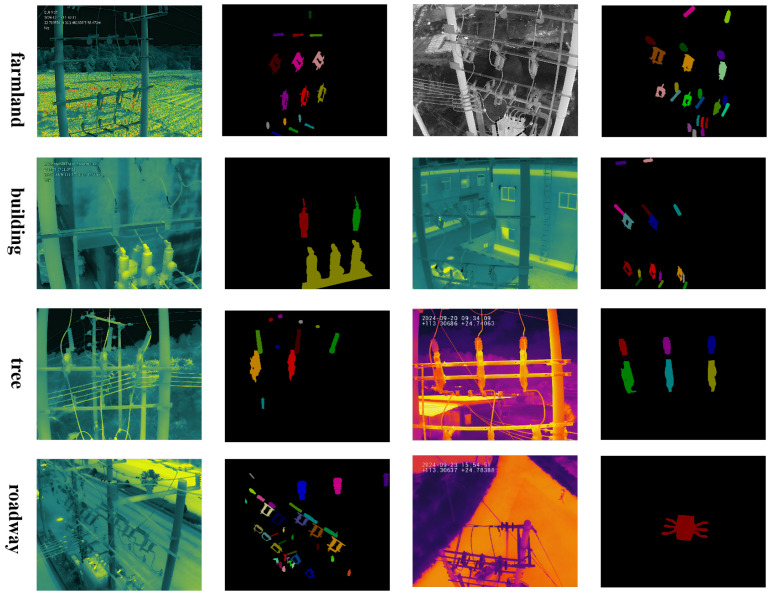
Examples of infrared images and instance masks from the constructed PDI dataset.

**Figure 6:**
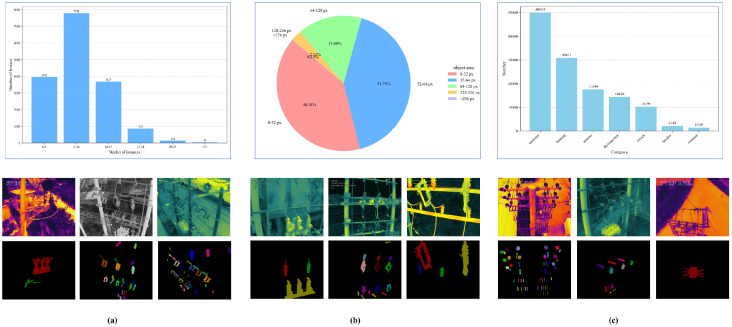
The challenges with the corresponding data samples of the constructed PDI dataset. Each power distribution equipment is considered as an instance. (**a**) Distribution imbalance. The number of instances in each infrared image varies considerably, with some having only 2 instances, while others have dozens of instances. (**b**) Scale imbalance. There are significant scale differences between instances of the same category and different categories, with some only occupying a few pixels, while others occupy hundreds of pixels. (**c**) Category imbalance. Instances of some categories appear frequently, while instances of other categories only appear a few times.

(1) Advantages:

- Largest scale of infrared images and instances: The PDI dataset contains 16,596 infrared images and 126,570 annotated instances, making it several times larger than existing infrared datasets in both image and annotation volume. This scale strongly meets the data requirements of modern data-driven methods and significantly improves the accuracy of instance segmentation for power distribution equipment.
- First UAV-based infrared dataset for power distribution inspection: While most existing infrared datasets focus on transmission lines or substations, PDI is the first to specifically target *power distribution inspection* using UAVs. Power distribution plays a critical role in delivering electricity to end-users, including households, industrial facilities, and commercial buildings, and is therefore essential for urban power grid reliability. Moreover, PDI includes seven critical categories of power distribution equipment, captured under real-world operating conditions.
- Rich scenarios and diverse instance patterns: Power distribution equipment exhibits significant variations in appearance. Different viewpoints introduce arbitrary orientations, partial deformations, and occlusions; different flight altitudes lead to drastic scale changes; and different inspection periods result in diverse thermal radiation distributions. In addition, PDI covers hundreds of geographical regions across Guangdong Province (e.g., Yunfu City, Dongguan City), where equipment is embedded in complex backgrounds such as trees, buildings, farmland, and roadways. Representative examples are shown in Figure 5.

(2) Challenges:

- Distribution imbalance: The number of instances per image varies considerably. Some images contain up to 49 instances, while others include only a single instance. The most common range is 5∼9 instances per image, whereas images with more than 30 instances are rare. This irregular distribution is illustrated in Figure 6a.
- Scale imbalance: Significant scale variations exist both within and across categories. For example, the largest switch measures 401 × 403 pixels (far exceeding the 96 × 96 reference, covering nearly 50% of the image area), while the smallest bushing is only 3 × 5 pixels (well below 32 × 32, less than 1% of the image area). Moreover, even within the same category, scale differences are dramatic: the largest bushing reaches 157 × 368 pixels (about 20% of the image area), whereas the smallest switch is only 7 × 15 pixels (less than 1% of the image area). Across categories, the variation is also evident, ranging from 32 × 32 pixels to 256 × 256 pixels, as shown in Figure 6b.
- Category imbalance: The frequency of categories is highly uneven. The most frequent category, insulator, contains 50,033 instances, while the least frequent, terminal, has only 1349 instances. This results in a long-tailed distribution with a ratio difference exceeding 30×, as illustrated in Figure 6c.

## 5. Experiment

### 5.1. Comparison Methods and Implementation Details

All comparison models are implemented on the MMDetection 3.1.0 platform with Python 3.8.19 and PyTorch 1.12.1. All experiments are conducted on an Ubuntu 22.04 LTS system equipped with an NVIDIA GeForce RTX 4090 GPU (24 GB).

A total of ten state-of-the-art models are evaluated, including six two-stage methods: Mask RCNN [13], Cascade RCNN [19], Mask2Former [34], PowerNet (Mask R-CNN) [8], SCNet [35], and DetectoRS [36]; and four one-stage methods: QueryInst [37], RTMDet [38], SOLOv2 [20], and YOLACT [21]. Each model is initialized with COCO-pretrained weights and trained with the default hyperparameter configurations provided by MMDetection.

The proposed framework (RE) is model-agnostic. To evaluate its compatibility and effectiveness, four representative modern methods are adopted as baselines: Mask RCNN, Cascade RCNN, SOLOv2, and YOLACT. By integrating them with the proposed framework, we obtain RE-Mask RCNN, RE-Cascade RCNN, RE-SOLOv2, and RE-YOLACT.

For optimization, stochastic gradient descent (SGD) with a momentum of 0.9 and a weight decay of 0.0001 is employed. The batch size is set to 8, and training is performed for 100 epochs. The initial learning rate is 0.02 and decays by a factor of 0.1 at the 50th and 75th epochs. Each infrared image is mapped to the RGB color space and normalized to the range [−1, 1]. Data augmentation includes random scaling, random translation, and random horizontal flipping with a probability of 0.5.

### 5.2. Evaluation Metrics

The standard COCO evaluation protocol [39] defines two primary metrics: detection average precision (${\mathrm{AP}}^{\mathrm{box}}$) and segmentation average precision (${\mathrm{AP}}^{\mathrm{seg}}$), which evaluate object localization and mask quality, respectively. AP is computed as the area under the precision–recall (PR) curve, where precision and recall are given by:

(19) $$\mathrm{Precision}=\frac{TP}{TP+FP},\;\mathrm{Recall}=\frac{TP}{TP+FN},$$

with TP, FP, and FN denote true positives, false positives, and false negatives. ${\mathrm{AP}}^{\mathrm{box}}$ measures the overlap between predicted and ground-truth bounding boxes, while ${\mathrm{AP}}^{\mathrm{seg}}$ measures the overlap between predicted and ground-truth masks. To ensure comprehensive evaluation, COCO averages AP across multiple IoU thresholds (0.5:0.95 with a step of 0.05).

In addition to AP, the F1-score is employed as a complementary metric that balances precision and recall:

(20) $$F1\text{-}\mathrm{score}=2\times \frac{\mathrm{Precision}\times \mathrm{Recall}}{\mathrm{Precision}+\mathrm{Recall}}.$$

Unlike AP, which integrates performance over all recall levels, the F1-score is computed at a specific operating point (e.g., IoU = 0.5). This makes it particularly useful in class-imbalanced scenarios, where a single balanced indicator of detection accuracy and completeness is needed.

### 5.3. Ablation Studies

To validate the effectiveness of the proposed framework, Mask RCNN [13] is adopted as the baseline, and the experimental results on the PDI dataset are presented in Table 2.

#### 5.3.1. Effect of ORD

The ORD is applied independently to generate reconstructed images, operating in parallel with the ISD. As shown in rows 1 and 2 of Table 2, RE-Mask RCNN achieves performance gains of 1.12% (${\mathrm{AP}}^{\mathrm{box}}$: 53.50 → 54.10) and 1.07% (${\mathrm{AP}}^{\mathrm{seg}}$: 46.80 → 47.30) compared to the original Mask RCNN. This improvement is attributed to the ORD reconstructing object regions, thereby enhancing the model’s ability to distinguish foreground objects from the background.

#### 5.3.2. Effect of DFE Module

The DFE module is applied in conjunction with ORD. As shown in rows 1 and 3 of Table 2, ${\mathrm{AP}}^{\mathrm{seg}}$ of RE-Mask RCNN improves by 0.43% compared to the baseline. By embedding the difference map as object features into the model, the DFE module strengthens the spatial features of objects. However, ${\mathrm{AP}}^{\mathrm{box}}$ decreases by 0.57%, likely due to background noise introduced during feature compensation.

#### 5.3.3. Effect of Filtration in DFE Module

**Figure 7:**
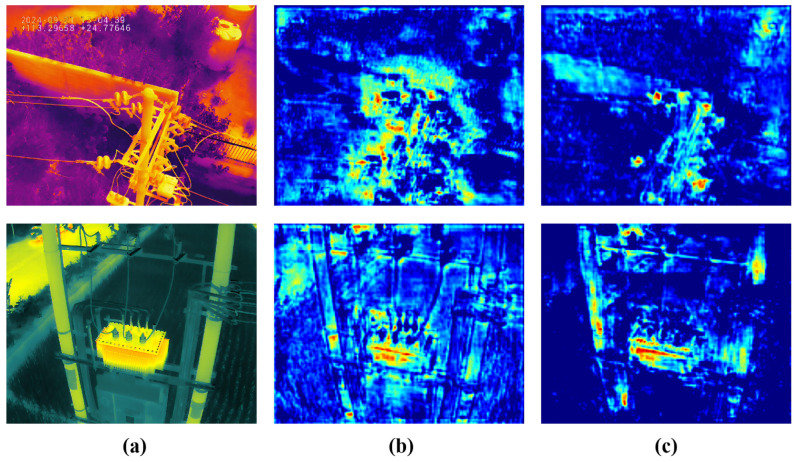
Visualization of filtration. (**a**) Input infrared image, (**b**,**c**) difference feature maps before and after filtration in the DFE module.

The pixel value differences between the reconstructed image and the input image produce varying degrees of activation in the difference image, with much of the noise potentially confusing the objects. To address this, a Gumbel-sigmoid function is employed for binary filtering while maintaining differentiability. As shown in rows 1 and 4 of Table 2, RE-Mask RCNN achieves performance gains of 3.18% (${\mathrm{AP}}^{\mathrm{box}}$: 53.50 → 55.20) and 2.56% (${\mathrm{AP}}^{\mathrm{seg}}$: 46.80 → 48.00) compared to the original Mask RCNN. As illustrated in Figure 7, the Gumbel-sigmoid function effectively suppresses incorrect activations caused by noise, allowing the model to preserve object-related features and thereby enhancing the reliability of object features.

### 5.4. Qualitative Results

**Figure 8:**
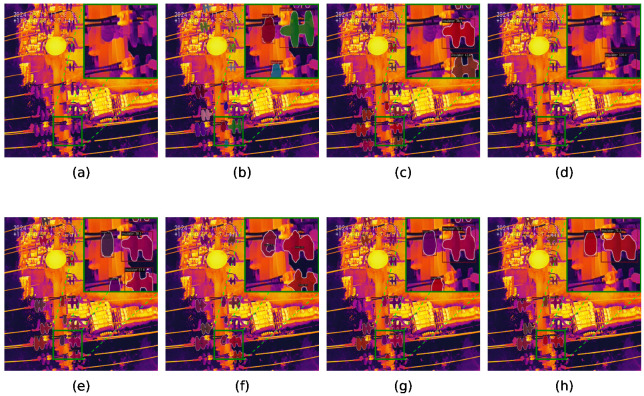

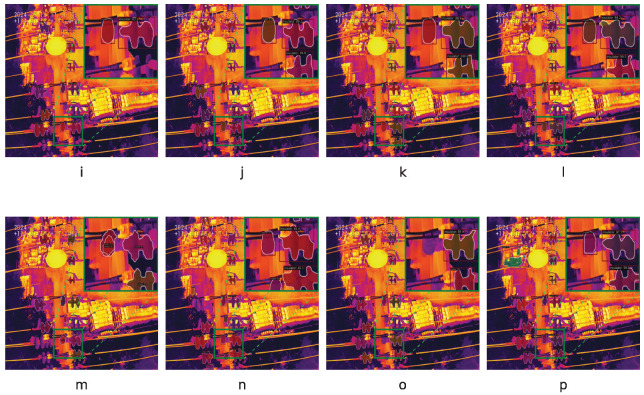
Instance segmentation results of insulators on the PDI dataset. (**a**) Input, (**b**) ground truth, (**c**) QueryInst, (**d**) Detectors, (**e**) Mask2Former, (**f**) RTMDet, (**g**) SCNet, (**h**) PowerNet, (**i**) Mask RCNN, (**j**) RE-Mask RCNN, (**k**) Cascade RCNN, (**l**) RE-Cascade RCNN, (**m**) SOLOv2, (**n**) RE-SOLOv2, (**o**) YOLOACT, (**p**) RE-YOLOACT.

**Figure 9:**
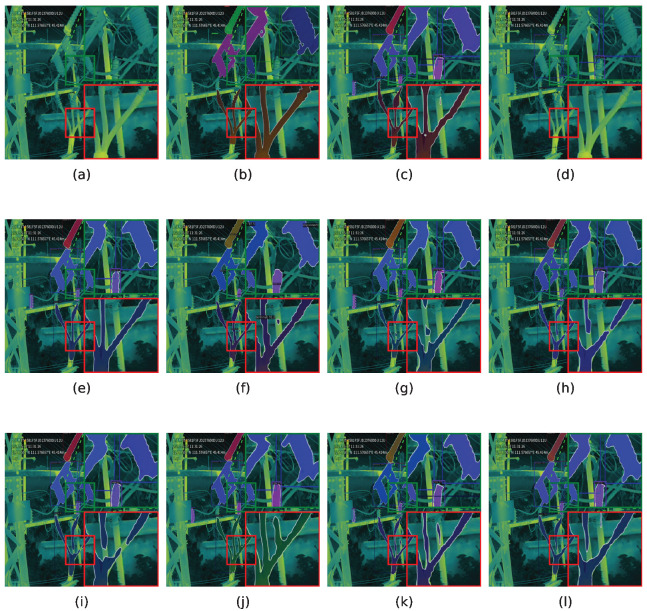

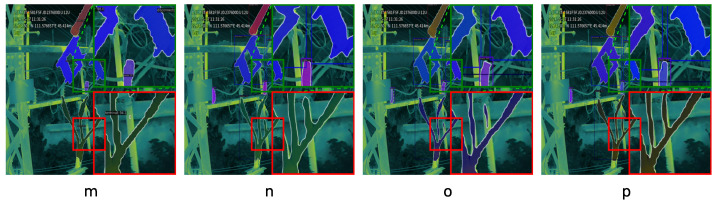
Instance segmentation results of disconnectors and terminal on the PDI dataset. (**a**) Input, (**b**) ground truth, (**c**) QueryInst, (**d**) Detectors, (**e**) Mask2Former, (**f**) RTMDet, (**g**) SCNet, (**h**) PowerNet, (**i**) Mask RCNN, (**j**) RE-Mask RCNN, (**k**) Cascade RCNN, (**l**) RE-Cascade RCNN, (**m**) SOLOv2, (**n**) RE-SOLOv2, (**o**) YOLOACT, (**p**) RE-YOLOACT.

The instance segmentation results of different methods are shown in Figure 8 and Figure 9, with highlighted regions in the upper-right and lower-right corners. Cascade RCNN [19] employs multi-stage threshold refinement to reduce missed recognition of small-scale insulators, while RTMDet [38] leverages dilated convolution to preserve object context, improving recognition of large-scale disconnectors. However, both methods struggle with precise edge localization. QueryInst [37] balances detection of small-scale insulators and large-scale disconnectors via object-level querying, but exhibits limitations in low-contrast scenarios, often failing to delineate edges clearly and occasionally incorporating background noise. Detectors [36] sometimes misrecognize terminals, SOLOv2 [20] may detect insulators repeatedly, and Mask RCNN [13] often fails to localize more than half of the terminals. In contrast, RE-Cascade RCNN accurately captures small-scale insulators with high confidence, and RE-SOLOv2 preserves the structural integrity of complex disconnectors, producing smooth and complete edges. Compared with the ground truth, the proposed framework consistently improves both object recognition and localization by suppressing information loss and enhancing the spatial features of objects.

### 5.5. Quantitative Results

The instance segmentation performance of different methods is reported in Table 3. The proposed framework demonstrates consistent improvements across both overall and single-category metrics. For example, RE-Cascade RCNN achieves ${\mathrm{AP}}^{\mathrm{box}}$ of 57.70 and ${\mathrm{AP}}^{\mathrm{seg}}$ of 47.70, surpassing the original Cascade RCNN by 3.78% and 1.71%, respectively. Similarly, RE-YOLOACT improves ${\mathrm{AP}}^{\mathrm{box}}$ to 52.50 and ${\mathrm{AP}}^{\mathrm{seg}}$ to 43.10, outperforming the original YOLOACT by 4.37% and 5.38%, respectively. For small-scale bushings, RE-Mask RCNN shows gains of 2.00% in ${\mathrm{AP}}^{\mathrm{box}}$ and 2.86% in ${\mathrm{AP}}^{\mathrm{seg}}$ compared to Mask RCNN. For insulators with complex edges, RE-Mask RCNN achieves improvements of 1.79% in ${\mathrm{AP}}^{\mathrm{box}}$ and 1.70% in ${\mathrm{AP}}^{\mathrm{seg}}$. These results indicate that the proposed framework effectively distinguishes diverse object categories and maintains high accuracy under challenging conditions, ranking first across multiple scenarios and demonstrating robust generalization capability.

### 5.6. Expand Experiments

#### 5.6.1. Category Imbalance

Category imbalance is a key challenge in the PDI dataset, affecting model performance. To address this, two common strategies are employed: category-balanced sampling (CBS) and loss re-weighting (LRW). CBS over-samples images from tail categories, such as terminal and arrester, often combined with data augmentation (e.g., random affine transformations) to expand diversity. LRW modifies the classification loss, for instance using focal loss [40] or weighting inversely proportional to category frequency, to emphasize tail categories without adding data.

Experiments are conducted using Mask RCNN [13] and RE-Mask RCNN, with results summarized in Table 4. For tail categories, CBS improves terminal ${\mathrm{AP}}^{\mathrm{seg}}$ from 11.70 to 13.50 and arrester ${\mathrm{AP}}^{\mathrm{box}}$ from 44.60 to 45.10, demonstrating enhanced localization without affecting major categories. LRW primarily boosts recognition, with terminal ${\mathrm{AP}}^{\mathrm{box}}$ rising from 41.00 to 42.40 and ${\mathrm{AP}}^{\mathrm{seg}}$ from 11.70 to 12.90, reflecting increased classification confidence.

Overall, RE-Mask RCNN with CBS achieves 55.80 ${\mathrm{AP}}^{\mathrm{box}}$ and 48.40 ${\mathrm{AP}}^{\mathrm{seg}}$, while RE-Mask RCNN with LRW attains 55.40 ${\mathrm{AP}}^{\mathrm{box}}$ and 48.30 ${\mathrm{AP}}^{\mathrm{seg}}$, both exceeding the baseline RE-Mask RCNN. These results indicate that CBS and LRW effectively mitigate tail category deficiencies, and RE-Mask RCNN consistently benefits from these strategies, providing a practical solution for class imbalance in power equipment instance segmentation.

#### 5.6.2. Scale Imbalance

The prevalence of small-scale objects is the primary manifestation of scale imbalance in the PDI dataset, affecting both inter-category size variation (e.g., switches are generally larger than bushings) and extreme intra-category variation (the largest and smallest bushings can differ by orders of magnitude). The proposed framework is specifically designed to address this challenge, particularly improving detection for small-scale objects.

Analysis of F1-score and recall metrics in Table 5 demonstrates that RE-based methods consistently enhance recall across most categories, reflecting increased sensitivity to positive samples. For instance, RE-Mask RCNN achieves an average recall of 84.33, outperforming the baseline Mask RCNN. Notable gains occur in terminal (+3.28) and arrester (+1.31), indicating improved detection of challenging objects. F1-score improvements are generally more modest but still positive; RE-Mask RCNN raises the F1-score for breaker from 92.08 to 92.63 and for arrester from 74.22 to 74.55, demonstrating better balance between precision and recall. RE-Cascade RCNN shows an average recall increase of 2.70, with a substantial gain of 4.80 for switch, though its F1-score slightly decreases from 81.80 to 81.70, suggesting a minor precision-recall trade-off. In contrast, RE-YOLOACT and RE-SOLOv2 exhibit marginal improvements, with categories such as insulator and disconnector showing near-baseline performance, implying limited gains from the framework in these architectures. Overall, the RE-based framework effectively enhances detection and feature representation for small-scale and challenging objects.

#### 5.6.3. Power Transmission Inspection

To verify the versatility and effectiveness of the proposed framework, experiments are conducted on the publicly available HRSeg dataset [8], which contains 7 categories of transmission equipment, including composite insulator, suspension clamp, C-style clamp, strain clamp, glass insulator, parallel groove clamp, and T-style clamp. The results are summarized in Table 6.

RE-Mask RCNN improves ${\mathrm{AP}}^{\mathrm{box}}$ and ${\mathrm{AP}}^{\mathrm{seg}}$ by 2.80 and 2.10 points, respectively, compared to Mask RCNN (51.70 vs. 48.90 and 41.60 vs. 39.50). Similarly, RE-YOLOACT achieves gains of 1.30 in ${\mathrm{AP}}^{\mathrm{box}}$ and 0.80 in ${\mathrm{AP}}^{\mathrm{seg}}$ (44.60 vs. 43.30 and 31.40 vs. 30.60). These improvements demonstrate that the proposed framework consistently enhances both single-category and full-category performance.

Overall, the results indicate that the framework is applicable to power transmission inspection and delivers favorable performance on both publicly available and the constructed PDI datasets.

## 6. Discussion

### 6.1. Framework Complexity Analysis

Table 7 reports the average running time (ART) and the number of parameters (Params) of different methods on the PDI dataset, with an input infrared image resolution of 640 × 512. The proposed framework is model-agnostic, allowing seamless integration into both two-stage and one-stage architectures. Although the ORD and DFE modules increase ART and Params compared to the baselines, the resulting accuracy improvements justify this overhead.

For example, RE-Mask RCNN adds 8.80 ms in ART and 1.04M parameters relative to Mask RCNN, while achieving gains of 3.18% and 2.56% in ${\mathrm{AP}}^{\mathrm{box}}$ and ${\mathrm{AP}}^{\mathrm{seg}}$. This trade-off highlights the advantage of enhancing object features without excessively compromising efficiency. Moreover, the framework maintains competitive ART and Params compared to other strong baselines. The efficiency is due to the linear spatial-temporal complexity of ORD and DFE, keeping computational costs manageable even in resource-constrained environments.

**Figure 10:**
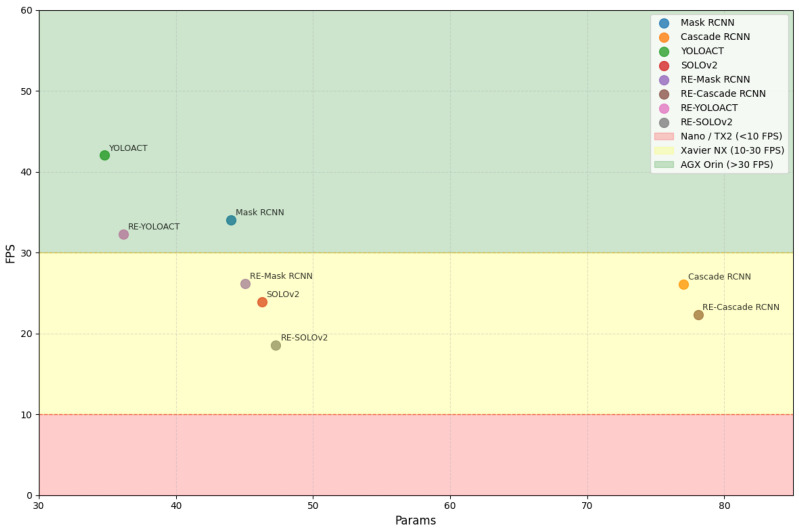
Instance segmentation models for NVIDIA Jetson platform suitability.

Furthermore, we contextualize Params and FPS relative to typical UAV onboard computing systems (e.g., NVIDIA Jetson platforms), as shown in Figure 10. Each model is positioned according to computational cost and achievable inference speed from Table 7. The background regions indicate the suitability ranges of different Jetson platforms: red (<10 FPS) for Jetson Nano/TX2, yellow (10–30 FPS) for Jetson Xavier NX, and green (>30 FPS) for Jetson AGX Orin.

YOLOACT and RE-YOLOACT occupy the upper-left region, providing a favorable trade-off between Params and FPS, making them suitable for real-time deployment on mid-power platforms such as Xavier NX. In contrast, heavier models like Cascade RCNN and RE-Cascade RCNN lie toward the right-bottom, requiring high-power platforms like AGX Orin for near real-time performance. These results are obtained without model pruning, quantization, or knowledge distillation, suggesting further potential for deployment on low-power platforms via model compression and optimization without substantial accuracy loss.

### 6.2. Visualization of the ORD and DFE Module

**Figure 11:**
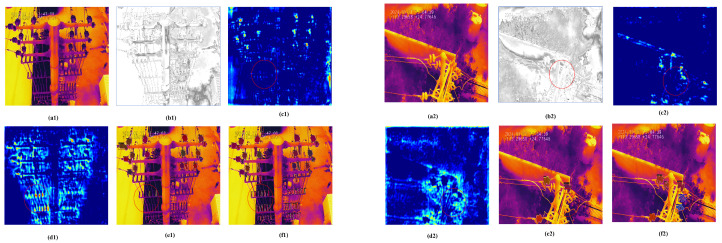
Visualization of the ORD and DFE module. (**a1**,**a2**) Input infrared image, (**b1**,**a2**) the difference image between the ORD result and the input infrared image. (**c1**–**d2**) feature maps before and after the DFE module. (**e1**–**f2**) instance masks of the baseline and the proposed method. Mask RCNN is selected as a baseline. We use red circles to highlight key regions in two examples, which are represented as 1 and 2, respectively.

Figure 11 visualizes the effects of the ORD and DFE modules, with Mask RCNN [13] as the baseline. Two representative challenges are highlighted: small-scale insulators on the left suffer from content compression, while disconnectors with complex structures on the right exhibit severe edge fragmentation. These issues lead to sparse backbone features, resulting in collapsed content for small insulators and fragmented edges for disconnectors. The difference image between the ORD reconstruction and the input clearly indicates the location and degree of information loss, with high-error regions corresponding to small objects and structural discontinuities. By integrating the ORD and DFE modules, object features are progressively reconstructed and refined: small insulator content is enhanced, and fractured edges of disconnectors are connected. Corresponding instance segmentation results demonstrate that the proposed framework achieves more accurate and structurally consistent predictions than the baseline.

### 6.3. Application Discussion

The proposed framework can be applied in two critical scenarios for preventing grid outages:

- High-Accuracy Preventive Maintenance Analytics: Designed for offline processing of UAV-captured images, this scenario prioritizes maximum accuracy over computational cost. Accurate instance segmentation of all power distribution equipment serves as the foundation for subsequent automated temperature diagnosis, supporting reliable preventive maintenance.
- Real-Time Onboard Screening: Although the current framework emphasizes accuracy, its model-agnostic nature allows integration with lightweight backbones (e.g., YOLOACT). Future work will focus on pruning and quantization for deployment on embedded platforms (e.g., NVIDIA Jetson), enabling UAVs to perform real-time preliminary screening and generate immediate alerts for overheating. As shown in Table 7, current models are unoptimized, indicating additional potential for low-power real-time deployment.

## 7. Conclusions

This paper explores the problem of information loss of objects and the limitations of existing methods. Therefore, an object reconstruction decoder is first proposed to replay the object features by reconstructing the object regions. Then, a difference feature enhancement module is proposed to adaptively complement the object features. They collaborate to improve the spatial feature representations of objects and show strong generalization and superiority on the constructed dataset PDI. This dataset is the first infrared dataset oriented to power distribution inspection, which has the advantages of large data volume, rich scenarios, and diverse object patterns, as well as the challenges of scale imbalance, distribution imbalance, and category imbalance, which significantly facilitate real-world power distribution inspection. The proposed framework provides an intuitive insight into the problem of scale imbalance. However, due to the imaging characteristics of infrared images, the objects generally have low contrast, which confuses them with the background. In future work, we will focus on the following directions:

- Multi-modal fusion: Infrared images often exhibit low contrast, limiting object discrimination. Fusing complementary information from visible images, which provide rich spatial textures and fine-grained details, can significantly improve detection and localization accuracy, particularly in complex scenarios.
- Semi-supervised and self-supervised learning: Annotating instance-level masks in infrared images is costly, restricting large-scale labeled datasets. Semi-supervised or self-supervised learning can exploit abundant unlabeled data, serving as effective pre-training strategies to learn generalizable and discriminative features while reducing reliance on labeled samples.
- Resource-efficient deployment: UAV-based applications require efficient processing under strict resource constraints. Future work will explore model compression and acceleration techniques, including pruning, quantization, knowledge distillation, and lightweight architectures, to enable real-world deployment while maintaining performance in dynamic aerial environments.

## Figures and Tables

**Table 1:** Comparison of existing infrared datasets and the constructed PDI dataset.

Dataset	Images	Instances	Categories	Power Industry	Annotation Level	Scenario Level	Type	Year
Wang et. al. [28]	1200	-	1	substation	instance	multiple	UAV	2020
Ma et. al. [2]	1626	-	4	distribution	instance	multiple	handheld	2020
Choi et. al. [29]	420	-	1	transmission	pixel	multiple	UAV	2022
Wang et. al. [3]	4839	14,005	17	substation	pixel	single	handheld	2022
Zhao et. al. [22]	400	563	4	substation	instance	single	UAV	2022
Zheng et. al. [30]	2760	8410	1	substation	object	multiple	handheld	2022
Zhou et. al. [14]	2540	-	1	transmission	instance	single	handheld	2023
Ou et. al. [31]	1263	245	5	substation	object	single	UAV	2023
Li et. al. [32]	1861	-	5	substation	object	multiple	UAV	2023
Xu et. al. [4]	600	-	4	transmission	pixel	multiple	UAV	2023
Xu et. al. [4]	400	-	4	substation	pixel	multiple	handheld	2023
Zhao et. al. [33]	874	1854	1	substation	instance	multiple	UAV	2024
Li et. al. [8]	11,415	45,145	2	transmission	instance	multiple	UAV	2025
**PDI (ours)**	**16,596**	**126,570**	7	**distribution**	**instance**	**various**	**UAV**	2025

(1) The scenario level is determined by the group of scenarios (geographical sites). In general, 1∼10 groups are considered as simple, 10∼100 groups are considered as multiple, and more than 100 groups are considered as various. (2) The categories only count parent categories.

**Table 2:** Performance of ablation studies. ORD: object reconstruction decoder, DFE: difference feature enhancement module, F: filtration in the DFE module. The symbol ✓ denotes the selected item.

ORD	DFE	F	Avg
			53.50/46.80
✓			54.10/47.30
✓	✓		53.20/47.00
✓	✓	✓	55.20/48.00

**Table 3:** ${\mathrm{AP}}^{\mathrm{box}}$ and ${\mathrm{AP}}^{\mathrm{seg}}$ performance evaluation of different methods on the constructed PDI dataset. The left value is ${\mathrm{AP}}^{\mathrm{box}}$ and the right value is ${\mathrm{AP}}^{\mathrm{seg}}$.

Method	bushing	insulator	disconnector	arrester	breaker	switch	terminal	Avg
QueryInst [37]	47.20/47.80	65.00/62.30	66.90/59.20	48.40/50.20	59.30/60.20	66.50/53.90	33.80/15.60	55.30/49.90
RTMDet [38]	51.00/45.10	70.00/60.90	68.50/56.30	49.60/44.90	60.70/57.20	68.70/48.10	33.60/15.80	57.40/46.90
SCNet [38]	53.90/55.80	71.00/68.80	69.60/64.30	53.80/56.30	67.00/67.90	69.00/59.90	44.30/21.60	61.20/56.40
DetectoRS [36]	47.30/-	65.00/-	65.40/-	46.50/-	69.20/-	64.40/-	45.70/-	57.60/-
Mask2Former [34]	55.10/57.60	72.20/70.50	69.80/66.00	55.60/58.70	70.50/70.30	71.20/61.50	46.90/21.60	63.00/58.00
PowerNet [8]	44.20/45.10	62.70/60.10	64.60/55.60	45.60/47.80	59.70/57.80	63.90/49.40	37.20/8.40	54.00/46.30
Mask RCNN [13]	44.90/45.50	61.50/58.90	62.90/55.20	43.30/45.80	63.90/62.10	60.40/48.90	37.90/11.50	53.50/46.80
RE-Mask RCNN	45.80/46.80	62.60/59.90	63.80/56.10	44.60/46.90	65.00/62.70	63.30/51.50	41.00/11.70	55.20/48.00
Cascade RCNN [19]	46.40/46.30	63.80/59.70	65.40/55.10	46.10/46.80	64.00/61.20	64.80/49.10	38.80/10.50	55.60/46.90
RE-Cascade RCNN	47.30/46.10	66.40/61.70	67.30/57.10	48.60/48.50	64.60/61.30	65.50/50.50	44.00/8.90	57.70/47.70
YOLOACT [21]	42.10/35.00	56.50/49.90	58.90/49.80	40.30/36.20	58.90/59.70	58.10/42.10	37.10/13.60	50.30/40.90
RE-YOLOACT	44.10/36.60	59.30/52.50	61.60/52.30	43.60/39.50	59.70/61.90	60.80/45.00	38.30/13.60	52.50/43.10
SOLOv2 [20]	-/31.30	-/51.20	-/55.90	-/39.00	-/68.20	-/46.20	-/24.10	-/45.10
RE-SOLOv2	-/33.60	-/54.70	-/58.40	-/41.70	-/68.50	-/49.00	-/26.20	-/47.50

Due to the design architecture, some methods do not have AP^box^ or AP^seg^ values, represented as ’-’.

**Table 4:** Comparison between two common category imbalance strategies. CBS: category-balanced sampling, LRW: loss re-weighting. The left value is AP^box^ and the right value is AP^seg^.

Method	bushing	insulator	disconnector	arrester	breaker	switch	terminal	Avg
Mask RCNN [13]	44.90/45.50	61.50/58.90	62.90/55.20	43.30/45.80	63.90/62.10	60.40/48.90	37.90/11.50	53.50/46.80
RE-Mask RCNN	45.80/46.80	62.60/59.90	63.80/56.10	44.60/46.90	65.00/62.70	63.30/51.50	41.00/11.70	55.20/48.00
Mask RCNN + CBS	45.20/45.20	61.70/59.10	63.20/55.40	43.80/46.30	64.10/62.30	60.90/49.60	40.50/14.10	54.20/47.40
RE-Mask RCNN + CBS	46.10/47.20	62.80/60.10	64.20/56.30	45.10/47.10	65.40/62.90	63.70/52.10	43.00/13.50	55.80/48.40
Mask RCNN + LRW	45.00/45.70	61.70/59.05	63.10/55.40	43.60/46.10	64.00/62.20	60.80/49.40	39.80/13.20	54.00/47.30
RE-Mask RCNN + LRW	45.80/46.80	62.10/60.10	64.00/56.30	44.90/47.30	65.20/62.70	63.60/51.90	42.40/12.90	55.40/48.30

**Table 5:** F1-score and recall performance evaluation of different methods on the constructed PDI dataset. The left value is the recall and the right value is the F1-score.

Method	bushing	insulator	disconnector	arrester	breaker	switch	terminal	Avg
Mask RCNN [13]	82.70/82.90	85.04/84.03	87.00/84.98	75.24/74.22	92.40/92.08	84.42/84.12	73.77/77.42	82.93/82.82
Cascade RCNN [19]	85.60/80.90	84.70/83.30	87.20/-	79.90/73.00	90.80/92.80	84.90/84.40	72.90/78.90	83.70/81.80
YOLOACT [21]	80.20/81.40	83.90/83.20	87.40/-	72.20/72.90	90.80/91.80	86.80/85.40	76.50/78.50	82.60/82.60
SOLOv2 [20]	80.20/81.40	83.90/83.20	87.40/85.10	72.20/72.90	90.80/91.80	86.80/85.40	76.50/78.50	82.60/82.60
RE-Mask RCNN	82.90/82.92	85.65/84.05	86.72/84.95	76.55/74.55	92.84/92.63	87.08/85.32	77.05/79.32	84.33/82.52
RE-Cascade RCNN	85.30/80.90	89.40/81.60	88.60/84.30	80.40/72.40	92.40/91.80	89.70/84.40	79.00/76.60	86.40/81.70
RE-YOLOACT	81.60/82.60	86.10/84.30	89.20/85.60	76.60/74.40	91.90/92.10	87.20/85.60	75.30/79.20	84.00/83.40
RE-SOLOv2	81.60/82.60	86.10/84.30	89.20/85.60	76.60/74.40	91.90/92.10	87.20/85.60	75.30/79.20	84.00/83.40

**Table 6:** AP^box^ and AP^seg^ performance evaluation of different methods on the public HRSeg dataset. The left value is ${\mathrm{AP}}^{\mathrm{box}}$ and the right value is AP^seg^.

Method	CI	SusC	CC	StrC	GI	PGC	TC	Avg
QueryInst [37]	65.20/37.90	52.50/49.80	19.20/24.70	48.40/30.80	68.60/53.60	28.30/35.70	70.20/53.80	50.40/40.90
RTMDet [38]	66.00/42.20	58.70/50.00	20.10/20.30	57.90/24.10	73.20/57.20	30.00/27.40	75.80/48.00	54.50/38.50
SCNet [35]	68.60/42.70	60.30/59.80	23.30/28.00	58.40/40.80	73.60/60.80	34.80/42.70	78.20/62.30	56.80/48.20
DetectoRS [36]	61.50/-	53.80/-	16.10/-	49.10/-	66.00/-	25.50/-	72.50/-	49.20/-
Mask2Former [34]	70.40/43.50	61.80/62.20	27.40/33.10	58.70/40.20	74.10/60.30	36.60/45.00	78.90/62.70	58.30/49.60
PowerNet [8]	63.70/37.30	52.00/50.80	19.90/23.30	50.50/31.20	69.00/54.40	28.10/35.10	72.30/54.50	50.80/40.90
Mask RCNN [13]	61.00/34.80	49.90/49.10	18.90/22.60	49.00/30.20	66.50/52.30	26.60/33.60	70.60/53.50	48.90/39.50
RE-Mask RCNN	63.50/37.00	53.00/51.60	20.20/24.70	52.10/31.70	70.10/55.20	29.40/36.10	73.60/54.90	51.70/41.60
Cascade RCNN [19]	63.20/35.90	52.80/49.80	16.20/20.30	49.90/30.30	67.90/52.50	25.50/33.00	72.20/52.80	49.60/39.20
RE-Cascade RCNN	64.70/36.80	53.30/50.90	20.20/24.40	52.20/30.80	70.60/54.70	28.90/36.70	74.20/54.80	52.00/41.30
YOLOACT [21]	48.60/35.20	48.40/41.40	15.10/16.40	41.70/9.80	57.60/48.60	25.90/22.10	65.80/40.40	43.30/30.60
RE-YOLOACT	51.10/37.50	49.30/42.10	17.30/17.40	43.70/10.40	58.70/49.30	25.70/22.50	66.40/40.60	44.60/31.40
SOLOv2 [20]	-/47.20	-/44.20	-/16.60	-/24.00	-/59.40	-/24.10	-/50.20	-/38.00
RE-SOLOv2	-/48.10	-/46.10	-/20.10	-/29.20	-/61.20	-/26.70	-/52.40	-/40.70

CI is composite insulator, SusC is suspension clamp, CC is C-style clamp, StrC is strain clamp, GI is glass insulator, PGC is parallel groove clamp, and TC is T-style clamp. Due to the design architecture, some methods do not have AP^box^ or AP^seg^ values, represented as ‘-’.

**Table 7:** Analysis of model complexity. ART: the average running time of each image, Params: the number of parameters, FPS: the frames per second.

Method	ART/ms	Param/M	Avg	FPS
Mask RCNN [13]	29.40	44.00	53.50/46.80	34.01
RE-Mask RCNN	38.20	45.04	55.20/48.00	26.18
Cascade RCNN [19]	38.30	77.04	55.60/46.90	26.11
RE-Cascade RCNN	44.80	78.08	57.70/47.70	22.32
SOLOv2 [20]	41.80	46.26	-/45.10	23.92
RE-SOLOv2	53.80	47.29	-/47.50	18.59
YOLOACT [21]	23.80	34.77	50.30/40.90	18.59
RE-YOLOACT	31.00	36.16	52.50/43.10	32.26

Due to the design architecture, some methods do not have AP^box^ or AP^seg^ values, represented as ‘-’.

## Data Availability

Data are contained within the article.
